# Tuberculosis drug discovery in the post-post-genomic era

**DOI:** 10.1002/emmm.201201772

**Published:** 2014-01-08

**Authors:** Benoit Lechartier, Jan Rybniker, Alimuddin Zumla, Stewart T Cole

**Affiliations:** 1 Ecole Polytechnique Fédérale de Lausanne, Global Health InstituteLausanne, Switzerland; 2 1st Department of Internal Medicine, University of CologneCologne, Germany; 3 Division of Infection and Immunity, University College London and UCLHospitals NHS Foundation TrustLondon, UK

**Keywords:** drug candidates, drug discovery, genomics, screening, tuberculosis

## Abstract

The expectation that genomics would result in new therapeutic interventions for infectious diseases remains unfulfilled. In the post-genomic era, the decade immediately following the availability of the genome sequence of *Mycobacterium tuberculosis*, tuberculosis (TB) drug discovery relied heavily on the target-based approach but this proved unsuccessful leading to a return to whole cell screening. Genomics underpinned screening by providing knowledge and many enabling technologies, most importantly whole genome resequencing to find resistance mutations and targets, and this resulted in a selection of leads and new TB drug candidates that are reviewed here. Unexpectedly, many new targets were found to be ‘promiscuous’ as they were inhibited by a variety of different compounds. In the post-post-genomics era, more advanced technologies have been implemented and these include high-content screening, screening for inhibitors of latency, the use of conditional knock-down mutants for validated targets and siRNA screens. In addition, immunomodulation and pharmacological manipulation of host functions are being explored in an attempt to widen our therapeutic options.

## Introduction

Genomics was supposed to revolutionize drug discovery and lead to numerous new therapeutic interventions. The use of bioinformatics for identifying potential drug targets that were restricted to selected pathogens, absent from humans and readily validated genetically was certainly appealing. In the area of anti-infectives, genomics has been largely disappointing as many of the rationally chosen new targets have transpired to be intractable, non-essential during disease or simply undruggable (Payne *et al*, 2007). Tuberculosis (TB) drug discovery has also suffered from the overoptimism inspired by the post-genomics revolution but, unlike other infectious disease areas, reasonable progress has been achieved nonetheless. Here, we will illustrate how genome-derived methods have underpinned TB drug discovery efforts, conducted mainly through whole-cell screening methodologies, and provided enabling technologies and valuable knowledge about the etiologic agent, *Mycobacterium tuberculosis* ( *Mtb*). The limitations of classical whole-cell screening have led to the creation of more sophisticated screens in the post-post-genomics era and these will be discussed in this review.

## Tuberculosis as a global health problem

With 8.7 million new active cases in 2011 and mortality of 1.4 million, TB remains an enormous global health problem (Zumla *et al*, 2013b). The success of *Mtb* is due to a perfect adaptation to the environment of its major host, the human macrophage. *Mtb* circumvents the bactericidal immune response and persists intracellularly after shutting down its own metabolism (Gengenbacher & Kaufmann, 2012). It is estimated that one-third of the world's population is afflicted by *Mtb* latent infection, meaning that viable but ‘dormant’ bacteria are contained within granulomas of these individuals although there is growing evidence for a spectrum of different bacterial physiological states contributing to subclinical TB (Robertson *et al*, 2012). Immune suppression leads to reactivation of the disease, explaining the high TB burden in people living with HIV and AIDS.

To avoid antibiotic resistance, it is mandatory to administer combination therapy comprising rifampicin, isoniazid, pyrazinamide and ethambutol for 2 months followed by 4 months of rifampicin and isoniazid to treat active TB. Despite this, drug resistance is an alarming problem and multi-drug resistant (MDR-TB) and extensively drug resistant strains (XDR-TB) are on the rise (Zumla *et al*, 2013b). Thus, the development and implementation of new drugs is a major keystone for future control of TB. The past decade has seen some success here since several promising compounds are in pre-clinical and clinical trials (Zumla *et al*, 2013a). Notably, in 2012, the diarylquinoline bedaquiline became the first FDA-approved anti-TB drug in more than 40 years (Zumla *et al*, 2013a).

The desirable attributes of a new TB drug are numerous and, ideally, include activity against both replicating and non-replicating bacteria, and penetration within tissues and granulomas — eventually leading to shortening of treatment duration. A new drug should have a novel target or mechanism of action allowing for treatment of MDR-and XDR-TB, and, most challengingly, be compatible with other anti-TB agents and drugs for treating comorbidities such as HIV/AIDS or diabetes. Patients co-infected with HIV-TB may receive >6 drugs including anti-HIV and anti-TB medications, thereby increasing the potential side-effects of drug-drug interactions and posing an enormous logistical challenge for the clinician.

Considerable effort has been made in recent years in the hunt for new medicines that would improve TB treatment and subsequent disease control. Four principal approaches have been followed: (i) discovering new drug candidates, (ii) repurposing existing antimicrobials, (iii) modulating host functions to increase cure rates, and (iv) developing innovative modes of drug administration. Only the first and third of these will be discussed in detail.

## TB drug research in the post-genomic era

Since 1998 when the first complete genome sequence of *Mtb* became available (Cole *et al*, 1998), genomics has provided a valuable drug discovery resource by catalyzing genetic manipulation and spawning powerful technologies including micro-array-based transcriptomics, proteomics, comparative genomics and structural genomics. These post-genomic advances impacted our understanding of *Mtb* biology considerably (Lew *et al*, 2011) and facilitated subsequent drug target identification and validation. However, many of the targets that were validated as being essential *in vitro* by genetic means, such as gene replacement or saturation transposon mutagenesis (Sassetti *et al*, 2003), proved not to be required during infection due to genetic redundancy or metabolic scavenging, leading us to appreciate that pharmacological target-validation was far more reliable and a better predictor of success.

**Figure 1:**
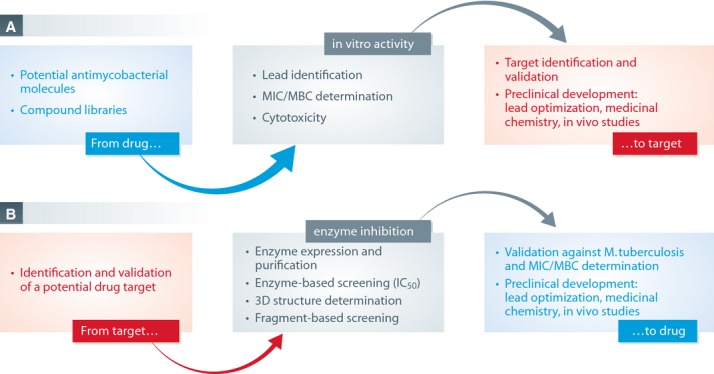
The figure displays the two main distinct methodologies in the search for new TB drugs. It is noteworthy that a target identified after a chemical screen (A) can enter the target-to-drug pipeline in enzyme-based screening (B). MIC, minimal inhibitory concentration; MBC, minimal bactericidal concentration; IC_50_, half maximal inhibitory concentration.

TB drug discovery can follow two main routes (Sala & Hartkoorn, 2011): the drug-to-target or the target-to-drug approaches (Fig 1). All the current drugs and candidates in clinical trials were derived from a drug-to-target path involving high throughput screening against whole cells, highlighting the importance of this approach. However, post-genomic tools now support the target-to-drug route and we will provide examples illustrating this area of research too.

In the late 90's whole genome sequencing (WGS) of bacteria was slow, extremely costly and labour-intensive. Nowadays, next generation sequencing platforms are widely available and WGS is used intensively to identify single nucleotide polymorphisms (SNP) or other mutations in the genome of spontaneous drug-resistant mutants allowing for target identification of novel compounds (Table 1). However, with some hit compounds generating resistant-*Mtb* mutants is not possible, making target identification and validation problematic. In these cases, post genomics tools have again been successfully used to give further insight into mechanism of action. For instance, using transcriptional profiling, before and after drug exposure, resulted in the identification of mycobacterial gene expression signatures that correlate with mechanism of action (Boshoff *et al*, 2004).

**Table 1:** Targets identified by whole genome sequencing

Target gene	Drug/Inhibitor	Sequencing technology	Reference
*atpE*	Bedaquiline (TMC207)	454	Andries *et al* (2005)
*ddn*	PA-824	NimbleGen	Manjunatha *et al* (2006)
*ddn*	Delamanid (OPC67683)	Not specified	Matsumoto *et al* (2006)
*dprE1*	BTZ043	ABI-Sanger	Makarov *et al* (2009)
*dprE1*	DNB1	ABI-Sanger	Christophe *et al* (2009)
*dprE1*	VI-9376	ABI-Sanger	Magnet *et al* (2010)
*dprE1*	377790	Illumina	Stanley *et al* (2012)
*dprE1*	TCA1	Illumina	Wang *et al* (2013)
*inhA*	Pyridomycin	Illumina	Hartkoorn *et al* (2012)
*mmpL3*	SQ109	Illumina	Tahlan *et al* (2012)
*mmpL3*	AU1253	SOLiD	Grzegorzewicz *et al* (2012)
*mmpL3*	THPP	Illumina	Remuinan *et al* (2013)
*mmpL3*	Spiro	Illumina	Remuinan *et al* (2013)
*mmpL3*	BM212	Illumina	La Rosa *et al* (2012)
*mmpL3*	C215	Illumina	Stanley *et al* (2012)
*qcrB*	Q203	Illumina	Pethe *et al* (2013)
*qcrB*	IP3	Illumina	Abrahams *et al* (2012b)

Although more elegant in terms of applied biology, the target-to-drug approach has been unsatisfactory mainly because converting the ability of a compound to inhibit a purified enzyme (IC_50_) into potency against whole cells (minimal inhibitory concentration: MIC) has proved a formidable obstacle to rational drug design. Promising enzyme inhibitors generally have poor, if any, activity against the bacterium itself, and this is presumably due to the complex mycobacterial cell envelope preventing uptake, to the action of efflux pumps or to compound inactivation. In addition, compound penetration alone does not always explain the lack of correlation between IC_50_ and MIC. For example, the level of protein inhibition needed to cause cidality is another important issue and can vary widely between targets (Wei *et al*, 2011).

Some of the clinically most advanced candidates are molecules with known MIC that were originally developed to treat other infectious diseases but have been repurposed for TB. These include compounds from the rifamycin, fluroquinolone and oxazolidinone classes, whose properties have been extensively reviewed elsewhere (Ma *et al*, 2010; Zumla *et al*, 2013a).

### How the classical drug-to-target pathway led to potential new TB drugs

Screening chemical libraries for compounds with MIC against live mycobacteria has delivered several promising TB drug candidates: the nitroimidazoles (PA-824 and delamanid), bedaquiline (TMC207), the newly identified imidazopyridine amide compound Q203, the diethylamine SQ109, and the benzothiazinone BTZ043 (Table 2).

**Table 2:** Main new chemical entities in development as antituberculosis drugs

Drug/Inhibitor	Class of drug	Hit identification strategy	Mechanism(s) of action	Mechanism(s) of resistance	Target(s) confirmed	Main reference(s)
PA-824	Nitroimidazoles	Whole-cell screening of metronidazole derivatives	Inhibition of cell wall synthesis and interference with cell respiration by NO production	Mutation in the nitroreductase Ddn required for pro-drug activation	No	Manjunatha *et al* (2006, 2009)
OPC-67683	Nitroimidazoles	Whole-cell screening for mycolic acid biosynthesis inhibitors	Inhibition of mycolic acid synthesis and NO production	Mutation in the nitroreductase Ddn required for pro-drug activation	No	Matsumoto *et al* (2006)
TMC207	Diarylquinoline	Whole-cell screening from quinolone derivatives	Inhibition of ATP biosynthesis	Mutation in the *c* subunit of Atp synthase, other(s)?	Yes	Andries *et al* (2005)
Q203	Imidazopyridine amide	Phenotypic screen in infected macrophages	Inhibition of the cytochrome *bc*_1_ complex	Mutation in the *b* subunit of the cytochrome *bc*_1_ complex	Yes	Pethe *et al* (2013)
SQ109	Diethylene diamine	Whole-cell screening of ethambutol derivatives	Inhibition of mycolic acid biosynthesis, other(s)?	Mutation in MmpL3	Yes	Protopopova *et al* (2005); Tahlan *et al* (2012)
BTZ043	Benzothiazinone	Whole-cell screening	Inhibition of arabinogalactan biosynthesis	Mutation in DprE1	Yes	Makarov *et al* (2009)

The related bicyclic nitroimidazoles, PA-824 and delamanid, are successes of the post-genomic era. Both nitroimidazoles are highly active against *Mtb* in aerobic conditions and against non-replicating or hypoxic bacteria (Stover *et al*, 2000; Matsumoto *et al*, 2006). Their mechanism of action was first thought to be due to inhibition of mycolic acid biosynthesis but is now known to be more complex. Manjunatha *et al* used microarray-based genome sequencing (Table 1) to identify mutations associated with resistance to PA-824 (Manjunatha *et al*, 2006). Interestingly, mutations affecting a nitroimidazo-oxazine specific nitroreductase, called Ddn (deazaflavin-dependent nitroreductase), confer resistance to both PA-824 and delamanid (Manjunatha *et al*, 2006). Ddn activates both these nitroimidazoles resulting in intracellular release of nitric oxide that presumably kills the bacteria (Singh *et al*, 2008). Gene expression profiling analysis after PA-824 exposure revealed activation of genes involved in cell wall synthesis as well as in respiration, suggesting that PA-824 indeed inhibits both pathways (Manjunatha *et al*, 2009).

In 2005, using a classical phenotypic screen, Andries *et al* (2005) discovered a very potent molecule, TMC207 or bedaquiline, an innovative, recently-approved drug candidate that offers considerable hope for curing MDR-TB cases. Through WGS, these investigators identified four missense mutations, associated with bedaquiline-resistance, in the *c* subunit of the mycobacterial ATP synthase encoded by *atpE* and then confirmed this as the drug target. However, a subsequent genetic study of spontaneously acquired TMC-207 resistant mutants revealed that only 15 out of 53 mutants harbored such mutations in *atpE* (Huitric *et al*, 2010) suggesting that the drug has either additional targets or resistance mechanisms, such as drug efflux. The discovery of bedaquiline highlighted energy metabolism in general and ATP synthase inhibition in particular as highly druggable.

Very recently, Pethe and colleagues further validated the proton-motive force and ATP synthesis as a major TB drug target with the discovery of a new class of imidazopyridine amide compounds that block growth of *Mtb* by targeting the respiratory cytochrome *bc*_1_ complex (Pethe *et al*, 2013). Using phenotypic high-content screening of infected macrophages (described in detail later), a series of compounds were selected and chemically optimized culminating in Q203, an inhibitor active at nanomolar concentrations with promising efficacy in murine TB models. Spontaneous-resistant mutants, subjected to WGS, harbor a single amino acid substitution in the cytochrome *b* subunit of the cytochrome *bc*_1_ complex associated with imidazopyridine amide-resistance. This innovative target is an essential component of the electron transport chain required for ATP synthesis and a second inhibitor of cytochrome *b* has also been reported (imidazo[1,2-*a*]pyridine (IP3); Table 1; Abrahams *et al*, 2012b).

The primary target of SQ109, a diethylamine drug candidate that inhibits mycolic acid synthesis (Protopopova *et al*, 2005), was only recently identified as MmpL3, a transmembrane transporter of trehalose monomycolate (Tahlan *et al*, 2012). Initially, the investigators were unable to identify spontaneous SQ109-resistant mutants; however, using ethylenediamine analogues, resistant mutants were generated that showed cross-resistance to SQ109 and WGS revealed mutations in the *mmpL3* gene. Interestingly, many other MmpL3 inhibitors have been found and amongst these are the adamantyl urea compound (AU1235), which is structurally related to SQ109 (Grzegorzewicz *et al*, 2012), and the benzimidazole C215 (Stanley *et al*, 2012). MmpL3 appears to be a ‘promiscuous’ target that is inhibited by diverse, chemically unrelated compounds. This is further exemplified by the recent discovery of BM212 (La Rosa *et al*, 2012) as well as tetrahydropyrazolo[1,5-a]pyrimidine-3-carboxamide and the spiro compounds (N-benzyl-6′,7′-dihydrospiro[piperidine-4,4′-thieno[3,2-c]pyran] analogues) that also target MmpL3 (Remuinan *et al*, 2013).

Benzothiazinones (BTZ), a new class of sulphur containing heterocyclic compounds, are extremely potent against *Mtb*, displaying low nanomolar bactericidal activity *in vitro* and in intracellular infection models (Makarov *et al*, 2009). Genetic and biochemical studies, as well as transcriptomic and proteomic analyses, identified the DprE1 subunit of the essential enzyme decaprenylphosphoryl-β-D-ribose 2′-epimerase as the target. Inhibition of DprE1 abolishes arabinogalactan synthesis, eventually leading to cell lysis. This actinobacteria-specific target was further validated when it was found to be inhibited by other nitro-aromatic molecules, dinitrobenzamides (Christophe *et al*, 2009), VI-9376 (Magnet *et al*, 2010) and the nitro-substituted triazole, 377 790 (Stanley *et al*, 2012), and by TCA1, a small molecule inhibitor with no nitro group (Wang *et al*, 2013). DprE1 also appears to be a promiscuous target.

### Rare examples of rational drug design — from target to drug

Despite the considerable effort invested, the target-based approach has had very limited impact on the discovery of novel TB drugs (Sala & Hartkoorn, 2011). Here, we discuss some of the more promising results obtained from structure-guided inhibitor design with two well-understood systems.

Ethionamide (ETH) is an approved second-line drug but with considerable side effects (Weinstein *et al*, 1962) that inhibits the enoyl-ACP reductase, InhA, the target of isoniazid (INH), thus preventing mycolic acid synthesis (Banerjee *et al*, 1994). ETH is a pro-drug activated by the mono-oxygenase EthA, whose expression is repressed by EthR (Baulard *et al*, 2000). Activation and, in turn, potency of the drug are limited by low constitutive levels of EthA. EthR inhibition leads to EthA overproduction and increases the activity of ETH against *Mtb*. Drug-like inhibitors of EthR, such as BDM31343, were designed using the crystal structure as a template and these boost ETH activity, improve its therapeutic index and triple its potency in mice (Willand *et al*, 2009).

The glyoxylate shunt is an important metabolic pathway for *Mtb* physiology, since it is upregulated in the absence of glycolysis to maintain the tricarboxylic acid cycle during the persistent phase of infection (McKinney *et al*, 2000). Mammals lack the two major enzymes in the pathway, isocitrate lyase and malate synthase (Kondrashov *et al*, 2006), making these enzymes promising and specific antibacterial drug targets. Many years were spent unsuccessfully trying to drug isocitrate lyase but progress was later made with malate synthase. Elegant structure-based drug design was used to develop potent phenyl-diketo acid inhibitors of malate synthase that are active in a mouse model of TB (Krieger *et al*, 2012). This study has chemically validated the glyoxylate pathway as a viable new drug target and identified a lead series of compounds for further preclinical development (Myler & Stacy, 2012).

### Screening natural products

Natural products, notably streptomycin and rifampicin, have played a considerable role in TB control. Despite the dominance of natural product antibiotics for other bacterial infections, there were no new natural product leads for mycobacteria until 2012 (Wright, 2012). Recently, Hartkoorn *et al* (Hartkoorn *et al*, 2012) deciphered the mechanism of action of pyridomycin, an antitubercular natural product first discovered in 1953 (Maeda *et al*, 1953). After selection of pyridomycin-resistant mutants followed by WGS, the authors identified the INH target InhA as the target of pyridomycin (Table 1). Since INH is a prodrug that requires activation by the catalase-peroxidase KatG, resistance is most frequently associated with mutations in the *katG* gene (Zhang & Yew, 2009). INH-resistant clinical isolates harboring a mutation in *katG* retained sensitivity to pyridomycin, making this natural product a promising lead compound to target InhA in drug-resistant isolates and providing encouragement for reinvestigation of natural product libraries using novel approaches (Garcia *et al*, 2012).

## Post-post-genomic screening strategies

Most whole-cell high throughput screens have been performed with medium containing glycerol as the main carbon source, thus poorly representing the environment *Mtb* encounters in the human host. This can give rise to disappointment as exemplified by a recent report (Pethe *et al*, 2010). Pyrimidine-imidazole derivatives with promising *in vitro* potency and desirable pharmacokinetic properties were identified using this medium but the hit compounds showed no efficacy *in vivo*. Mechanism of action studies revealed the hits to be active only in the glycerol-rich screening medium but not during host infection where glycerol metabolism is less relevant (Pethe *et al*, 2010). Thus, screening against *Mtb* under intracellular conditions may yield hits with better *in vivo* activity and care should also be taken in choosing the medium and mycobacterial species used for the orthologous *in vitro* screen.

### High content screening

High content screening using *Mtb*-infected macrophages exploits automated confocal fluorescence microscopy to quantify intracellular mycobacteria expressing GFP in a 384-well format (Christophe *et al*, 2009; Brodin & Christophe, 2011). This technique has several advantages: (i) Putative hit compounds will target proteins that are essential for intracellular survival which brings the screen closer to the *in vivo* setting; (ii) Compounds with intracellular activity are selected; (iii) Cytotoxic compounds with antimicrobial activity are eliminated which saves time and money. Using this technique, Christophe *et al* screened 57 000 compounds for growth inhibition of *Mtb* H37Rv-GFP in Raw264.7 macrophages (Christophe *et al*, 2009). Several clusters of active molecules were identified. Among these were the dinitrobenzamides (DNB1 and 2), discussed above, that are inhibitors of DprE1, the BTZ-target. The screen identified additional scaffolds with chemical structures unrelated to known antibacterials which are currently under investigation. Among them was Q203, a potent imidazopyridine amide derivative (Pethe *et al*, 2013).

### Screening against non-replicating bacteria

Yet another tactic to mimic *in vivo* conditions is screening under low oxygen conditions. In granulomatous lesions, mycobacteria are exposed to hypoxia which triggers a hypometabolic state leading to a non-replicative persistence phase (Gengenbacher & Kaufmann, 2012). These dormant bacteria are tolerant to many antimycobacterials, which is one reason for the lengthy duration of TB treatment. Persisting mycobacteria have a reduced but still significant ATP-pool which has to be maintained at low levels during dormancy (Mak *et al*, 2012). A screen was developed to target this pool under hypoxic conditions eventually thereby identifying compounds with activity against non-replicating *Mtb* (Mak *et al*, 2012). 600 000 compounds were screened against oxygen depleted *Mycobacterium bovis*-BCG leading to the identification of 32 clusters of active compounds that significantly reduce ATP-levels and show good MIC against replicating and non-replicating mycobacteria.

*Mtb* harbors many proteins that are essential only during intracellular infection and their characterization increases the number of putative targets for future targeted drug screens. Bryk *et al* screened for inhibitors of the dihydrolipoamide acyltransferase (DlaT), an enzyme used by the bacterium to resist host derived nitric oxide reactive oxygen intermediates (Bryk *et al*, 2008). Chemical screening for inhibitors of *Mtb* DlaT identified rhodanines as compounds that kill almost exclusively non-replicating mycobacteria in synergy with host immunity.

A simple approach to finding latency inhibitors is provided by the streptomycin-dependent 18b strain of *Mtb* because, when streptomycin is absent, 18b cannot grow and its metabolism shifts towards latency (Sala *et al*, 2010). Streptomycin-starved 18b (SS18b) has been used successfully for medium thoughput screens with more extensive screening still in progress (Sala *et al*, 2010). A major advantage of the SS18b model is its application to different animal models thus allowing information about efficacy against latency to be obtained *in vivo*. Using the SS18b system anti-TB drugs and leads have been ranked and cell wall inhibitors shown to have no efficacy in murine models whereas the drugs bedaquiline and rifapentine performed exceptionally well (Zhang *et al*, 2012).

### Target-based whole-cell screening

Target-based HTS and whole-cell HTS have their pros and cons. Thus, efforts were made to combine both methods for the selection of new compounds active against known targets. These target-based whole-cell screens make use of tetracycline-repressible mycobacterial promoters that allow the titrated expression of putative target proteins (Ehrt *et al*, 2005). Reducing the levels of an essential protein may sensitize the bacterium towards an inhibitor that acts on the down-regulated protein whereas wild-type bacteria should show a higher MIC for the same compound allowing for hit identification. Using this system, Abrahams *et al* generated conditional *Mtb* knock-down mutants of the essential *panC* gene, which encodes pantothenate synthase (Abrahams *et al*, 2012a). PanC is involved in the biosynthesis of pantothenate (vitamin B5) and the respective mutant is a pantothenate auxotroph and attenuated in mice. Conditionally lowering PanC levels sensitized the bacteria towards a set of PanC inhibitors identified using fragment-based approaches (Hung *et al*, 2009; Abrahams *et al*, 2012a). A subsequent compound library screen identified several flavones that showed 16-fold higher activity against the *panC*-knockdown mutant compared to the wild-type strain. Addition of pantothenate to the growth medium abrogated this effect. Though the compounds showed no inhibition of affinity-purified PanC in biochemical assays, it is likely that other enzymes of the pantothenate pathway or pantothenate-requiring enzymes are affected; this greatly reduces the number of putative targets to be examined. Target-based whole-cell screening promises to facilitate target validation for hit-compounds identified by conventional whole-cell screens.

### Cellular and animal models

The use of surrogate cells and mammalian hosts to mimick human disease is fraught with difficulties so, while there is no commonly accepted ‘gold standard’, pragmatism is required. For the purpose of cell-based screening it is critical that the cells behave in a robust and reproducible manner. Consequently, human or murine cell lines such as THP-1 or Raw264.7, respectively, tend to be favored over primary cells. Testing hit compounds for efficacy in animal models is generally determined by the quantities of compound available meaning that smaller animals, such as the mouse, are preferred. A wide range of animal models exist that replicate some but not all facets of human TB and the choice of species depends largely on the question to be addressed (Young, 2009). With respect to drug discovery and development the mouse again figures prominently but it should be remembered that while drug safety can be assessed in animal models drug efficacy is best established in humans.

## Manipulation of host functions

As an intracellular pathogen, *Mtb* exerts massive influence on the gene expression of its host. More than 5000 genes are differentially expressed in the lungs of *Mtb*-infected rabbits (Subbian *et al*, 2011). In phagocytes, many of these genes are dedicated to an effective immune response for clearing the pathogen yet *Mtb* is able to attenuate this host defense. Several siRNA screens have successfully identified specific host genes and the underlying mechanisms involved in dampening the microbicidal response (Kuijl *et al*, 2007; Jayaswal *et al*, 2010; Kumar *et al*, 2010). Intriguingly, small molecules can now be developed to abrogate the *Mtb*-induced alteration of host cell-signaling in order to boost an effective response of the immune system.

### Antimicrobial drugs that target host protein kinases

**Figure 2:**
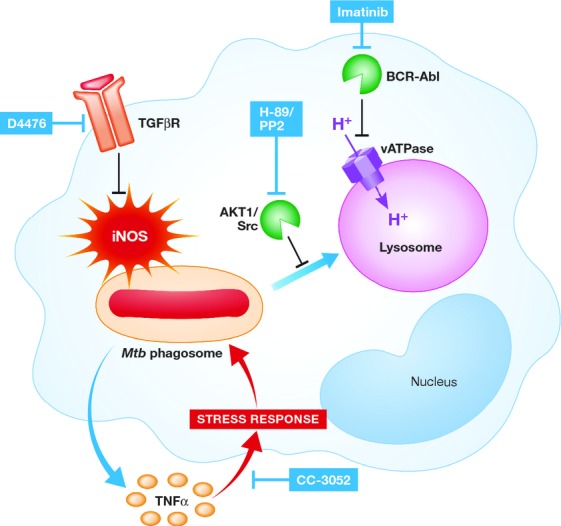
*Mtb* infection leads to the phosphorylation of AKT1 and upregulation of the Src-kinase which inhibits phago-lysosomal fusion. Subsequently, the AKT-inhibitor H-89 as well as the Src-kinase inhibitor PP2 promotes fusion of the *Mtb* phagosome with the lysosome. In addition, infected macrophages release TGF-β resulting in decreased levels of nitric oxide synthase (iNOS) most likely through phosphorylation of the TGF-β receptor (TGFβR). Blockage of this receptor, for example with the compound D4476, releases nitric oxide leading to enhanced clearance of the bacteria. Several pathogens (e.g. *Shigella* spp, *Salmonella* spp, *Yersinia* spp) are known to use the Abl-tyrosine-kinase to dampen a successful antimicrobial host response. In *Mtb* infected macrophages, blockage of this kinase with imatinib promotes acidification of the lysosome through up-regulation of the proton pumping enzyme vacuolar-type H^+^-adenosine triphosphatase (vATPase) resulting in improved intracellular killing of *Mtb*. Infected macrophages release the pro-inflammatory cytokine TNF-α. Though this cytokine promotes an antimycobacterial macrophage response, it also provokes a stress response in the bacteria with upregulation of the Dos-regulon facilitating the transition to metabolically inactive bacteria and persistence. Blockage of TNF-α by the compound CC-3052 alleviates this stress and may render the bacterial cells more susceptible to antimycobacterials such as isoniazid.

Using siRNAs targeting kinases and phosphatases of mouse macrophages, 11 host enzymes were identified whose depletion led to a reduction of the intracellular burden of various *Mtb* isolates (Jayaswal *et al*, 2010). Among these enzymes, the TGF-β type-1 receptor (TGFβRI), a serine threonine kinase, was further confirmed as a critical mediator for intracellular survival of *Mtb* by depletion of its ligand (TGF-β) and by the finding that the bacterial burden in *Mtb*-infected mice was lowered after treatment with D4476, a TGFβRI inhibitor. TGF-β was confirmed as an attractive TB target in a second, independent study where the lungs of chronically infected mice were treated with siRNA targeting TGF-β1; this led to a modest decrease of bacterial burden in C57/BL6 mice and, to a greater degree, in IL-10 knock-out mice (Rosas-Taraco *et al*, 2011). Interestingly, depletion of TGF-β, which inhibits the cell-mediated immunity stemming from the Th1 response during mycobacterial infections, increased expression of the bactericidal nitric oxide synthase (iNOS) and nitric oxide levels (Fig 2).

Another siRNA screen targeting the human kinome identified AKT1 as a central mediator for survival and growth of intracellular pathogens (Kuijl *et al*, 2007). Macrophage derived AKT1 is strongly phosphorylated during bacterial infections and several AKT1 inhibitors were shown to control intracellular replication of mycobacteria. Kujil *et al* also performed siRNA-based pathway analysis identifying a kinase network downstream of AKT1 as essential for phagosomal maturation during infections with *Salmonella typhimurium* and *Mtb*. This strategy may identify more target-specific kinase inhibitors, acting downstream of AKT1, to control intracellular pathogens (Fig 2).

Yet another approach to identify regulatory networks of intracellular pathogens is global gene expression profiling of the infected host. Using this methodology, a Src tyrosine kinase was shown to be a central regulator of *Mtb* intracellular survival (Karim *et al*, 2011). Key to this finding was a comparative microarray study of macrophages infected with the fully virulent strain H37Rv and the attenuated mutant H37Ra for up to 90 h. Src kinase was downregulated and dephosphorylated during infection with H37Ra but upregulated or unchanged during infection with the wild-type strain. Subsequently, treatment of H37Rv-infected macrophages with the Src-family kinase inhibitor PP2 promoted phagolysosomal fusion and significantly reduced the intracellular bacterial load *in vitro*.

Obviously, targeting innate immune signaling pathways may be a risky way to eradicate pathogens capable of causing severe and acute systemic infections. A therapeutic agent needs to be fine-tuned to conserve a functional immune response yet counter pathogen induced immune modeling *throughout all* stages of infection. The cancer drug and BCR-Abl kinase inhibitor Imatinib (Gleevec®, Novartis, Basel, Switzerland) seems to fulfill most of these prerequisites. A large number of viral and bacterial pathogens are known to use the Abl-tyrosine kinase family to regulate pathogenicity (Lebeis & Kalman, 2009). Treatment with Imatinib, which shows a remarkably low toxicity profile during long term treatment of chronic myelogenous leukemia patients (CML), was beneficial in the control of poxvirus infections in mice (Reeves *et al*, 2005). Recently, two independent studies have shown that the Abl tyrosine kinase is a regulator of mycobacterial pathogenesis in macrophages and Imatinib treatment reduces intracellular survival of *Mtb in vitro* (Napier *et al*, 2011; Bruns *et al*, 2012). Abl tyrosine kinase influences trafficking of lysosomes and blockage of the kinase significantly increased the acidification of intracellular vesicles, most likely by up-regulating the proton-pumping vacuolar-type ATPase (vATPase; Bruns *et al*, 2012). Subsequently, bactericidal proteases which are dependent on an acidic environment show enhanced activity leading to better control of intracellular *Mtb*. Since Imatinib treatment reduced bacterial burden in the lungs of *Mtb*-infected mice, further evaluation of this FDA-approved drug is warranted (Napier *et al*, 2011; Fig 2).

### Controlling cytokines in the infected host

The bactericidal role of pro-inflammatory cytokines in the *Mtb*-infected host is well established. Under certain clinical settings however, the pleiotropic effects of cytokines such as TNF-α can lead to a hyperinflammatory response, massive tissue damage and disease aggravation (Roca & Ramakrishnan, 2013). The immune reconstitution inflammatory syndrome (IRIS) that appears shortly after initiating anti-retroviral therapy in HIV-TB co-infected patients requires therapeutic interventions to abrogate the noxious effect of TNF-α. CC-3025, a phosphodiesterase-4 inhibitor and thalidomide analogue, is a potent inhibitor of TNF-α production. Interestingly, co-treatment of *Mtb*-infected rabbits with CC-3052 increases the bactericidal activity of INH (Subbian *et al*, 2011). An extensive transcriptomic study performed on both infected lung tissue and the pathogen revealed a possible mechanism for the observed synergy. It was shown that: (i) lung infection with *Mtb* leads to massive induction of host genes, including several in the TNF-α network and TNF-α itself; (ii) in turn *Mtb* upregulates the general stress response and dormancy ( *dosR*) regulons; (iii) INH-treatment promotes additional upregulation of the genes for the drug target, InhA, the activator, KatG, and other proteins involved in INH resistance; (iv) co-treatment with CC-3052 reduces cytokine expression levels, including TNF-α, which alleviates the bacterial stress response and, most interestingly, reduces expression levels of INH-induced genes. Thus CC-3052 treatment leads to bacterial cells which are metabolically active and more susceptible towards antibiotics.

There is also growing evidence that severity of disease and hyperinflammation is not only mediated by strain-specific virulence but also by host-encoded susceptibility (Ganachari *et al*, 2012; Tobin *et al*, 2012). SNPs found in the human leukotriene A_4_ hydrolase (LTA4H) locus illustrate the importance of a balanced pro-and anti-inflammatory response towards *Mtb*. LTA4H catalyzes the production of leukotriene B_4_, an eicosanoid that induces pro-inflammatory cytokines such as TNF-α. SNPs negatively affecting enzyme activity lead to an impaired immune response, more severe disease and higher mortality (Tobin *et al*, 2010). However, promoter polymorphisms positively affecting LTA4H expression increase inflammation leading to higher mortality in patients with tuberculous meningitis—a severe form of the disease that requires adjunctive anti-inflammatory treatment with glucocorticoids. Interestingly, patients with TB-meningitis who are homozygous for *LTA4H* promoter mutations may primarily benefit from adjuvant corticosteroid therapy (Tobin *et al*, 2012). This finding shows that knowledge of the patients' genotype not only provides information about susceptibility towards the disease, but may also allow tailored therapies for subsets of patients. This is also of major interest in the light of several clinical studies on adjunct immunotherapy with thalidomide, IL-2 or *Mycobacterium vaccae,* which failed to show significant beneficial effects for the patients (Schoeman *et al*, 2004; Uhlin *et al*, 2012). Deeper knowledge of the genetic background of patients enrolled in future studies may result in more favorable results.

**Pending issues**

Translating IC_50_ into MIC.

Designing whole-cell screens to avoid promiscuous targets.

Developing an algorithm to predict *in vivo* efficacy.

Combining candidate drugs with existing agents while ensuring compatibility with anti-retroviral drugs and simplifying clinical trials.

Minimizing potential side effects of host modifying agents, especially in HIV/TB co-infection or when used as adjunct therapy.

Better understanding of host-pathogen interaction is required to design targeted drugs, to optimize drug penetrance and to manipulate non-productive host responses.

Another SNP that influences the outcome of patients under certain circumstances is the *t* allele of the *Taq*I vitamin D receptor polymorphism. Though this non-synonymous SNP has no influence on vitamin D serum levels, a double-blind randomized controlled trial revealed that homozygous carriers showed faster sputum culture conversion when vitamin D_3_ was co-administered during the intensive-phase-treatment of pulmonary TB (Martineau *et al*, 2011). An ongoing series of clinical trials may identify additional patient subsets that could benefit from vitamin D_3_ substitution. Vitamin D deficiency is common in patients with active TB and the immunomodulatory effects of supplementation are pleiotropic and hard to interpret (Coussens *et al*, 2012). Bedside to bench research has since revealed that the IFN-γ-mediated antimycobacterial response of human macrophages is highly dependent on sufficient vitamin D_3_ serum levels (Fabri *et al*, 2011). Incubation of human monocytes with vitamin D-deficient serum abolished the IFN-γ dependent induction of antimicrobial peptides and blocked autophagolysosomal fusion thereby causing uncontrolled growth of intracellular bacilli. These effects were reversed when the cells were treated with vitamin D-sufficient serum.

## Concluding remarks

Research performed during the post-genomic era has successfully generated leads and candidate drugs for the treatment of TB. A reasonable drug development pipeline has arisen and there are grounds for cautious optimism provided that attrition of the current candidates can be managed, new leads are optimized and funding is sustained to ensure that their development is completed. Intriguingly, several promiscuous targets were uncovered during the post-genomic era and it is not clear if this were due to inherent properties of the proteins themselves or to the screening conditions and compound libraries used. There is hope that the more diverse technological approach to screening currently being applied will deliver additional targets, expand the compound classes and provide further drug candidates to ensure that the TB drug pipeline remains filled and gains in robustness. Although intellectually appealing and supported by a growing number of successes, targeting host functions is not without its problems. Foremost amongst these, of course, is toxicity due to the off-target effects of such inhibitors. However, in the context of MDR-TB, there may well be a case for their compassionate use together with an optimized background regime.

## Abbreviations

Cidality: the ability or power to kill microrganisms
Compound penetration: the ability or power of a drug to penetrate into tissues
Cytokine: proteins or peptides involved in cell signaling. Usually involved in the immune response and immune modulation
Gene replacement: replacing the copy of the gene originally present with an altered version
Granuloma: a focal, compact collection of inflammatory cells in which various types of immune cells surround an undegradable product (pathogen, foreign bodies or hypersensitivity reaction). The center of granulomas due to *Mtb* often contains necrotic material
Latent infection: presence of *Mtb* without symptoms and signs of active disease
Lysosome: acidic cell organelles that break down cellular debris and foreign particles such as bacteria
Phagosome: a cellular compartment which is formed out of the cell membrane when foreign bodies such as bacteria are taken up by a phagocytic cell (e.g. a macrophage)
Phenotypic screen: a drug screen that results in an altered phenotype of an organism or cell e.g. growth inhibition or death
Promiscuous target: A single target to which multiple pharmacophores bind but not necessarily at the same site
Protein kinases: enzymes that posttranslationally modify proteins through phosphorylation
Rational drug design: drug-like molecules are designed or selected for the modulation of a specific and known target (usually an enzyme) using structural information
Repurposed drugs: drugs against molecules developed and marketed for one clinical condition that are used for another
Single nucleotide polymorphism (SNP): change of a single nucleotide in genomic DNA. If the affected region codes for a protein, SNPs can lead to amino acid changes. If it lies in the promoter region it may increase or decrease gene expression
Targeted drug screen: uses a validated target to find inhibitors
Transposon mutagenesis: Insertion of a transposon into a gene in order to inactivate it and possibly confer a mutant phenotype
Whole-cell high throughput screen: trial and error screening of huge libraries of small molecule on cultured bacteria or infected cells using robotics
Whole genome sequencing (WGS): deciphering the complete DNA sequence of an organism. Used to find SNPs an other mutations

## References

[b1] Abrahams GL, Kumar A, Savvi S, Hung AW, Wen S, Abell C, Barry CE, Sherman DR, Boshoff HI, Mizrahi V (2012a). Pathway-selective sensitization of *Mycobacterium tuberculosis* for target-based whole-cell screening. Chem Biol.

[b2] Abrahams KA, Cox JA, Spivey VL, Loman NJ, Pallen MJ, Constantinidou C, Fernandez R, Alemparte C, Remuinan MJ, Barros D (2012b). Identification of novel imidazo[1,2-a]pyridine inhibitors targeting *M. tuberculosis* QcrB. PLoS One.

[b3] Andries K, Verhasselt P, Guillemont J, Gohlmann HW, Neefs JM, Winkler H, Van Gestel J, Timmerman P, Zhu M, Lee E (2005). A diarylquinoline drug active on the ATP synthase of *Mycobacterium tuberculosis*. Science.

[b4] Banerjee A, Dubnau E, Quemard A, Balasubramanian V, Um KS, Wilson T, Collins D, de Lisle G, Jacobs WR (1994). inhA, a gene encoding a target for isoniazid and ethionamide in *Mycobacterium tuberculosis*. Science.

[b5] Baulard AR, Betts JC, Engohang-Ndong J, Quan S, McAdam RA, Brennan PJ, Locht C, Besra GS (2000). Activation of the pro-drug ethionamide is regulated in mycobacteria. J Biol Chem.

[b6] Boshoff HI, Myers TG, Copp BR, McNeil MR, Wilson MA, Barry CE (2004). The transcriptional responses of *Mycobacterium tuberculosis* to inhibitors of metabolism: novel insights into drug mechanisms of action. J Biol Chem.

[b7] Brodin P, Christophe T (2011). High-content screening in infectious diseases. Curr Opin Chem Biol.

[b8] Bruns H, Stegelmann F, Fabri M, Dohner K, van Zandbergen G, Wagner M, Skinner M, Modlin RL, Stenger S (2012). Abelson tyrosine kinase controls phagosomal acidification required for killing of *Mycobacterium tuberculosis* in human macrophages. J Immunol.

[b9] Bryk R, Gold B, Venugopal A, Singh J, Samy R, Pupek K, Cao H, Popescu C, Gurney M, Hotha S (2008). Selective killing of nonreplicating mycobacteria. Cell Host Microbe.

[b10] Christophe T, Jackson M, Jeon HK, Fenistein D, Contreras-Dominguez M, Kim J, Genovesio A, Carralot JP, Ewann F, Kim EH (2009). High content screening identifies decaprenyl-phosphoribose 2′ epimerase as a target for intracellular antimycobacterial inhibitors. PLoS Pathog.

[b11] Cole ST, Brosch R, Parkhill J, Garnier T, Churcher C, Harris D, Gordon SV, Eiglmeier K, Gas S, Barry CE (1998). Deciphering the biology of *Mycobacterium tuberculosis* from the complete genome sequence. Nature.

[b12] Coussens AK, Wilkinson RJ, Hanifa Y, Nikolayevskyy V, Elkington PT, Islam K, Timms PM, Venton TR, Bothamley GH, Packe GE (2012). Vitamin D accelerates resolution of inflammatory responses during tuberculosis treatment. Proc Natl Acad Sci USA.

[b13] Ehrt S, Guo XV, Hickey CM, Ryou M, Monteleone M, Riley LW, Schnappinger D (2005). Controlling gene expression in mycobacteria with anhydrotetracycline and Tet repressor. Nucleic Acids Res.

[b14] Fabri M, Stenger S, Shin DM, Yuk JM, Liu PT, Realegeno S, Lee HM, Krutzik SR, Schenk M, Sieling PA (2011). Vitamin D is required for IFN-gamma-mediated antimicrobial activity of human macrophages. Sci Transl Med.

[b15] Ganachari M, Guio H, Zhao N, Flores-Villanueva PO (2012). Host gene-encoded severe lung TB: from genes to the potential pathways. Genes Immun.

[b16] Garcia A, Bocanegra-Garcia V, Palma-Nicolas JP, Rivera G (2012). Recent advances in antitubercular natural products. Eur J Med Chem.

[b17] Gengenbacher M, Kaufmann SH (2012). *Mycobacterium tuberculosis*: success through dormancy. FEMS Microbiol Rev.

[b18] Grzegorzewicz AE, Pham H, Gundi VA, Scherman MS, North EJ, Hess T, Jones V, Gruppo V, Born SE, Kordulakova J (2012). Inhibition of mycolic acid transport across the *Mycobacterium tuberculosis* plasma membrane. Nat Chem Biol.

[b19] Hartkoorn RC, Sala C, Neres J, Pojer F, Magnet S, Mukherjee R, Uplekar S, Boy-Rottger S, Altmann KH, Cole ST (2012). Towards a new tuberculosis drug: pyridomycin—nature's isoniazid. EMBO Mol Med.

[b20] Huitric E, Verhasselt P, Koul A, Andries K, Hoffner S, Andersson DI (2010). Rates and mechanisms of resistance development in *Mycobacterium tuberculosis* to a novel diarylquinoline ATP synthase inhibitor. Antimicrob Agents Chemother.

[b21] Hung AW, Silvestre HL, Wen S, Ciulli A, Blundell TL, Abell C (2009). Application of fragment growing and fragment linking to the discovery of inhibitors of *Mycobacterium tuberculosis* pantothenate synthetase. Angew Chem Int Ed Engl.

[b22] Jayaswal S, Kamal MA, Dua R, Gupta S, Majumdar T, Das G, Kumar D, Rao KV (2010). Identification of host-dependent survival factors for intracellular *Mycobacterium tuberculosis* through an siRNA screen. PLoS Pathog.

[b23] Karim AF, Chandra P, Chopra A, Siddiqui Z, Bhaskar A, Singh A, Kumar D (2011). Express path analysis identifies a tyrosine kinase Src-centric network regulating divergent host responses to *Mycobacterium tuberculosis* infection. J Biol Chem.

[b24] Kondrashov FA, Koonin EV, Morgunov IG, Finogenova TV, Kondrashova MN (2006). Evolution of glyoxylate cycle enzymes in Metazoa: evidence of multiple horizontal transfer events and pseudogene formation. Biol Direct.

[b25] Krieger IV, Freundlich JS, Gawandi VB, Roberts JP, Gawandi VB, Sun Q, Owen JL, Fraile MT, Huss SI, Lavandera JL (2012). Structure-guided discovery of phenyl-diketo acids as potent inhibitors of *M. tuberculosis* malate synthase. Chem Biol.

[b26] Kuijl C, Savage ND, Marsman M, Tuin AW, Janssen L, Egan DA, Ketema M, van den Nieuwendijk R, van den Eeden SJ, Geluk A (2007). Intracellular bacterial growth is controlled by a kinase network around PKB/AKT1. Nature.

[b27] Kumar D, Nath L, Kamal MA, Varshney A, Jain A, Singh S, Rao KV (2010). Genome-wide analysis of the host intracellular network that regulates survival of *Mycobacterium tuberculosis*. Cell.

[b28] La Rosa V, Poce G, Canseco JO, Buroni S, Pasca MR, Biava M, Raju RM, Porretta GC, Alfonso S, Battilocchio C (2012). MmpL3 is the cellular target of the antitubercular pyrrole derivative BM212. Antimicrob Agents Chemother.

[b29] Lebeis SL, Kalman D (2009). Aligning antimicrobial drug discovery with complex and redundant host-pathogen interactions. Cell Host Microbe.

[b30] Lew JM, Kapopoulou A, Jones LM, Cole ST (2011). TubercuList–10 years after. Tuberculosis.

[b31] Ma Z, Lienhardt C, McIlleron H, Nunn AJ, Wang X (2010). Global tuberculosis drug development pipeline: the need and the reality. Lancet.

[b32] Maeda K, Kosaka H, Okami Y, Umezawa H (1953). A new antibiotic, pyridomycin. J Antibiot.

[b33] Magnet S, Hartkoorn RC, Szekely R, Pato J, Triccas JA, Schneider P, Szantai-Kis C, Orfi L, Chambon M, Banfi D (2010). Leads for antitubercular compounds from kinase inhibitor library screens. Tuberculosis.

[b34] Mak PA, Rao SP, Ping Tan M, Lin X, Chyba J, Tay J, Ng SH, Tan BH, Cherian J, Duraiswamy J (2012). A high-throughput screen to identify inhibitors of ATP homeostasis in non-replicating *Mycobacterium tuberculosis*. ACS Chem Biol.

[b35] Makarov V, Manina G, Mikusova K, Mollmann U, Ryabova O, Saint-Joanis B, Dhar N, Pasca MR, Buroni S, Lucarelli AP (2009). Benzothiazinones kill *Mycobacterium tuberculosis* by blocking arabinan synthesis. Science.

[b36] Manjunatha U, Boshoff HI, Barry CE (2009). The mechanism of action of PA-824: novel insights from transcriptional profiling. Commun Integr Biol.

[b37] Manjunatha UH, Boshoff H, Dowd CS, Zhang L, Albert TJ, Norton JE, Daniels L, Dick T, Pang SS, Barry CE (2006). Identification of a nitroimidazo-oxazine-specific protein involved in PA-824 resistance in *Mycobacterium tuberculosis*. Proc Natl Acad Sci USA.

[b38] Martineau AR, Timms PM, Bothamley GH, Hanifa Y, Islam K, Claxton AP, Packe GE, Moore-Gillon JC, Darmalingam M, Davidson RN (2011). High-dose vitamin D(3) during intensive-phase antimicrobial treatment of pulmonary tuberculosis: a double-blind randomised controlled trial. Lancet.

[b39] Matsumoto M, Hashizume H, Tomishige T, Kawasaki M, Tsubouchi H, Sasaki H, Shimokawa Y, Komatsu M (2006). OPC-67683, a nitro-dihydro-imidazooxazole derivative with promising action against tuberculosis in vitro and in mice. PLoS Med.

[b40] McKinney JD, Honer zu Bentrup K, Munoz-Elias EJ, Miczak A, Chen B, Chan WT, Swenson D, Sacchettini JC, Jacobs WR, Russell DG (2000). Persistence of *Mycobacterium tuberculosis* in macrophages and mice requires the glyoxylate shunt enzyme isocitrate lyase. Nature.

[b41] Myler PJ, Stacy R (2012). A new drug for an old bug. Chem Biol.

[b42] Napier RJ, Rafi W, Cheruvu M, Powell KR, Zaunbrecher MA, Bornmann W, Salgame P, Shinnick TM, Kalman D (2011). Imatinib-sensitive tyrosine kinases regulate mycobacterial pathogenesis and represent therapeutic targets against tuberculosis. Cell Host Microbe.

[b43] Payne DJ, Gwynn MN, Holmes DJ, Pompliano DL (2007). Drugs for bad bugs: confronting the challenges of antibacterial discovery. Nat Rev Drug Discovery.

[b44] Pethe K, Bifani P, Jang J, Kang S, Park S, Ahn S, Jiricek J, Jung J, Jeon HK, Cechetto J (2013). Discovery of Q203, a potent clinical candidate for the treatment of tuberculosis. Nat Med.

[b45] Pethe K, Sequeira PC, Agarwalla S, Rhee K, Kuhen K, Phong WY, Patel V, Beer D, Walker JR, Duraiswamy J (2010). A chemical genetic screen in *Mycobacterium tuberculosis* identifies carbon-source-dependent growth inhibitors devoid of in vivo efficacy. Nat Commun.

[b46] Protopopova M, Hanrahan C, Nikonenko B, Samala R, Chen P, Gearhart J, Einck L, Nacy CA (2005). Identification of a new antitubercular drug candidate, SQ109, from a combinatorial library of 1,2-ethylenediamines. J Antimicrob Chemother.

[b47] Reeves PM, Bommarius B, Lebeis S, McNulty S, Christensen J, Swimm A, Chahroudi A, Chavan R, Feinberg MB, Veach D (2005). Disabling poxvirus pathogenesis by inhibition of Abl-family tyrosine kinases. Nat Med.

[b48] Remuinan MJ, Perez-Herran E, Rullas J, Alemparte C, Martinez-Hoyos M, Dow DJ, Afari J, Mehta N, Esquivias J, Jimenez E (2013). Tetrahydropyrazolo[1,5-a]pyrimidine-3-carboxamide and N-benzyl-6′,7′-dihydrospiro[piperidine-4,4′-thieno[3,2-c]pyran] analogues with bactericidal efficacy against *Mycobacterium tuberculosis* targeting MmpL3. PLoS One.

[b49] Robertson BD, Altmann D, Barry C, Bishai B, Cole S, Dick T, Duncan K, Dye C, Ehrt S, Esmail H (2012). Detection and treatment of subclinical tuberculosis. Tuberculosis.

[b50] Roca FJ, Ramakrishnan L (2013). TNF dually mediates resistance and susceptibility to mycobacteria via mitochondrial reactive oxygen species. Cell.

[b51] Rosas-Taraco AG, Higgins DM, Sanchez-Campillo J, Lee EJ, Orme IM, Gonzalez-Juarrero M (2011). Local pulmonary immunotherapy with siRNA targeting TGFbeta1 enhances antimicrobial capacity in *Mycobacterium tuberculosis* infected mice. Tuberculosis.

[b52] Sala C, Dhar N, Hartkoorn RC, Zhang M, Ha YH, Schneider P, Cole ST (2010). Simple model for testing drugs against nonreplicating *Mycobacterium tuberculosis*. Antimicrob Agents Chemother.

[b53] Sala C, Hartkoorn RC (2011). Tuberculosis drugs: new candidates and how to find more. Future Microbiol.

[b54] Sassetti CM, Boyd DH, Rubin EJ (2003). Genes required for mycobacterial growth defined by high density mutagenesis. Mol Microbiol.

[b55] Schoeman JF, Springer P, van Rensburg AJ, Swanevelder S, Hanekom WA, Haslett PA, Kaplan G (2004). Adjunctive thalidomide therapy for childhood tuberculous meningitis: results of a randomized study. J Child Neurol.

[b56] Singh R, Manjunatha U, Boshoff HI, Ha YH, Niyomrattanakit P, Ledwidge R, Dowd CS, Lee IY, Kim P, Zhang L (2008). PA-824 kills nonreplicating *Mycobacterium tuberculosis* by intracellular NO release. Science.

[b57] Stanley SA, Grant SS, Kawate T, Iwase N, Shimizu M, Wivagg C, Silvis M, Kazyanskaya E, Aquadro J, Golas A (2012). Identification of novel inhibitors of *M. tuberculosis* growth using whole cell based high-throughput screening. ACS Chem Biol.

[b58] Stover CK, Warrener P, VanDevanter DR, Sherman DR, Arain TM, Langhorne MH, Anderson SW, Towell JA, Yuan Y, McMurray DN (2000). A small-molecule nitroimidazopyran drug candidate for the treatment of tuberculosis. Nature.

[b59] Subbian S, Tsenova L, O'Brien P, Yang G, Koo MS, Peixoto B, Fallows D, Dartois V, Muller G, Kaplan G (2011). Phosphodiesterase-4 inhibition alters gene expression and improves isoniazid-mediated clearance of *Mycobacterium tuberculosis* in rabbit lungs. PLoS Pathog.

[b60] Tahlan K, Wilson R, Kastrinsky DB, Arora K, Nair V, Fischer E, Barnes SW, Walker JR, Alland D, Barry CE (2012). SQ109 targets MmpL3, a membrane transporter of trehalose monomycolate involved in mycolic acid donation to the cell wall core of *Mycobacterium tuberculosis*. Antimicrob Agents Chemother.

[b61] Tobin DM, Roca FJ, Oh SF, McFarland R, Vickery TW, Ray JP, Ko DC, Zou Y, Bang ND, Chau TT (2012). Host genotype-specific therapies can optimize the inflammatory response to mycobacterial infections. Cell.

[b62] Tobin DM, Vary JC, Ray JP, Walsh GS, Dunstan SJ, Bang ND, Hagge DA, Khadge S, King MC, Hawn TR (2010). The lta4 h locus modulates susceptibility to mycobacterial infection in zebrafish and humans. Cell.

[b63] Uhlin M, Andersson J, Zumla A, Maeurer M (2012). Adjunct immunotherapies for tuberculosis. J Infect Dis.

[b64] Wang F, Sambandan D, Halder R, Wang J, Batt SM, Weinrick B, Ahmad I, Yang P, Zhang Y, Kim J (2013). Identification of a small molecule with activity against drug-resistant and persistent tuberculosis. Proc Natl Acad Sci USA.

[b65] Wei JR, Krishnamoorthy V, Murphy K, Kim JH, Schnappinger D, Alber T, Sassetti CM, Rhee KY, Rubin EJ (2011). Depletion of antibiotic targets has widely varying effects on growth. Proc Natl Acad Sci USA.

[b66] Weinstein HJ, Hallett WY, Sarauw AS (1962). The absorption and toxicity of ethionamide. Am Rev Respir Dis.

[b67] Willand N, Dirie B, Carette X, Bifani P, Singhal A, Desroses M, Leroux F, Willery E, Mathys V, Deprez-Poulain R (2009). Synthetic EthR inhibitors boost antituberculous activity of ethionamide. Nat Med.

[b68] Wright GD (2012). Back to the future: a new ‘old’ lead for tuberculosis. EMBO Mol Med.

[b69] Young D (2009). Animal models of tuberculosis. Eur J Immunol.

[b70] Zhang M, Sala C, Hartkoorn RC, Dhar N, Mendoza-Losana A, Cole ST (2012). Streptomycin-starved *Mycobacterium tuberculosis* 18b, a drug discovery tool for latent tuberculosis. Antimicrob Agents Chemother.

[b71] Zhang Y, Yew WW (2009). Mechanisms of drug resistance in *Mycobacterium tuberculosis*. Int J Tuberc Lung Dis.

[b72] Zumla A, Nahid P, Cole ST (2013a). Advances in the development of new tuberculosis drugs and treatment regimens. Nat Rev Drug Discovery.

[b73] Zumla A, Raviglione M, Hafner R, von Reyn CF (2013b). Tuberculosis. New Engl J Med.

